# La toxocarose peut-elle être responsable d’une pancréatite aigue?

**DOI:** 10.11604/pamj.2018.29.180.9630

**Published:** 2018-03-28

**Authors:** Najah Boussetta, Bilel Arfaoui, Leila Metoui, Rim Dhahri, Bassem Louzir, Imen Gharsallah, Faida Ajili

**Affiliations:** 1 Service de Médecine Interne, Hôpital Militaire de Tunis, Tunisie

**Keywords:** Hyperéosinophilie, pancréatite aigüe, sérologie, toxocarose, traitement, Hypereosinophilia, acute pancreatitis, serology, toxocariasis, treatment

## Abstract

La toxocarose humaine est une zoonose parasitaire cosmopolite causée par *Toxocara canis* et *Toxocara catis*qui sont des ascarides des chiens et des chats. C’est une affection le plus souvent bénigne. Nous rapportons un cas de pancréatite aigue dans le décours une toxocarose.

## Abstract (en)

Human toxocariasis is a cosmopolitan parasitic zoonosis caused by Toxocara canis and Toxocara cati which are roundworms of dogs and cats. It is mostly a benign condition. We here report a case of acute pancreatitis secondary to toxocariasis.

## Introduction

La toxocarose humaine est une zoonose parasitaire cosmopolite causée par *Toxocara canis* et *Toxocara catis* qui sont des ascaridés des chiens et des chats. La transmission est orofécale et la contamination chez l’homme se fait après consommation d’aliments souillés par des excréments, ou lorsque les mains ont été en contact avec de la terre ou du sable souillé [1]. La toxocarose est une affection le plus souvent bénigne, voire asymptomatique. Les manifestations les plus fréquentes sont l’asthénie, la fièvre, l’hépato-splénomégalie, la bronchite et l’atteinte cutanée à type de prurigo et d’urticaire. Les atteintes graves sont rares notamment, cardiaques, neurologiques et oculaires. A notre connaissance, l’atteinte pancréatique n’a pas été décrite dans la littérature. Nous rapportons un cas de pancréatite aigue dans le décours une toxocarose.

## Patient et observation

Il s’agit d’un patient âgé de 24 ans suivi depuis 2 ans pour une hypertension artérielle traitée par de l’amlodipine. Il était hospitalisé dans notre service pour des épigastralgies transfixiantes, irradiantes vers le dos et associées à des vomissements bilieux d’apparition brutale. Il se plaignait également d’une diarrhée non glairo-sanglante. L'examen à l’admission trouvait un patient conscient et apyrétique. L'abdomen était souple et dépressible avec une sensibilité épigastrique et de l’hypochondre gauche sans défense. Par ailleurs l'examen cardio-vasculaire et respiratoire était sans particularité. A la biologie, la numération de la formule sanguine a montré une hyperleucocytose à 17200 éléments/mm^3^ avec des éosinophiles à 7900/mm^3^ contrôlés à 2 reprises, une hémoglobine 16,2 g/dl et des plaquettes à 284000/mm^3^. La CRP était à 29 mg/l et le bilan hépatique était normal. Par ailleurs la lipasémie et l’amylasémie étaient élevées respectivement à 624 UI/l (< 190UI/l) et à 453 UI/l (< 92UI/l). La radiographie du thorax était sans anomalie. Le scanner abdominal a objectivé une pancréatite stade D selon la classification de Balthazar avec présence d’une collection liquidienne au niveau de l’espace para-rénal antérieur gauche. Les voies biliaires et le canal de Wirsung étaient libres. L’anamnèse n’a pas révélé de prise médicamenteuse ni de traumatisme et le patient n’était pas éthylique. Le bilan lipidique et la calcémie étaient sans anomalie (calcémie à 2,22mmol/l, cholestérolémie à 3,3mmol/l, triglycérides à 0,81mmol/l). La conduite à tenir était de prescrire une diète absolue pendant 3 jours et surveiller le patient. L’évolution était marquée par la persistance des douleurs abdominales et de la diarrhée. Devant l’hyperéosinophilie massive un examen parasitologique des selles qui était négatif. Les sérologies du kyste hydatique, de l’anguillulose, de l’ankylostomiase, de la distomatose étaient négatives. Devant la notion d’élevage occasionnel de chiens à domicile, on a complété par la sérologie de la toxocarose qui était positive par la technique ELISA et Western blot. Le diagnostic de toxocarose pancréatique a été retenu et le patient a été traité par l’albendazole a la dose de 10 mg /kg/j pendant 15 jours. L'évolution était favorable avec disparition des douleurs épigastriques et de la diarrhée et la diminution du taux des polynucléaires éosinophiles à 400/mm^3^. L’amlodipine n’a pas été arrêtée.

## Discussion

La pancréatite aiguë est une maladie inflammatoire du pancréas, caractérisée par l’autodigestion du parenchyme pancréatique résultant d’une activation intracellulaire inappropriée des enzymes pancréatiques protéolytiques [1]. Le diagnostic positif est suggéré par la présence d’au moins deux de ces éléments: une douleur abdominale, au minimum un triplement des concentrations sériques d’amylase et/ou de lipase et des résultats d’imagerie caractéristiques de la pancréatite aiguë [2]. Notre patient avait les 3 éléments confirmant le diagnostic de pancréatite aigue. Les étiologies des pancréatites sont multiples dont l’éthylisme et les lithiases biliaires qui constituent les causes les plus fréquentes. Les causes les moins fréquentes comprennent: l’hypertriglycéridémie, l’hypercalcémie, les infections virales (oreillons, Coxsackie), les médicaments (azathioprine, mercaptopurine, didanosine), le dysfonctionnement du sphincter d’Oddi, les tumeurs, les traumatismes, la chirurgie, la cholangio-pancréatographie rétrograde endoscopique (CPRE) et les anomalies congénitales (pancréas divisum, pancréas annulaire, cholédochocèle, duplication kystique du duodénum). La pancréatite aiguë peut être idiopathique dans près de 20% des cas. Les étiologies parasitaires sont dominées par les parasites intestinaux notamment l’ascaris [3 ,4]. En revoyant la littérature, le pancréas ne semble pas être un site classique de migration du *Toxocara*, à la différence du foie et du tube digestif. En effet, chez l’adulte la toxocarose prend deux formes, la forme asymptomatique ou occulte résultant d’une infestation minime dont le diagnostic est fait à l’occasion d’une hyperéosinophilie et la forme mineure due à une infestation modérée, associant des signes non spécifiques: douleurs abdominales, asthénie persistante, atteintes cutanées, rhumatologiques, pulmonaires [5, 6]. Des manifestations oculaires, cardiaques et neurologiques ont été aussi décrites [7]. Le diagnostic de certitude repose sur la sérologie. Les techniques utilisées sont principalement de type immunoenzymatique (Elisa) avec une sensibilité de l’ordre de 90%. Leur spécificité n’est pas parfaite car il y’a souvent des réactions croisées avec d’autres parasites comme les nématodoses surtout chez les patients résidents dans les zones tropicales. La technique Western blot est une approche qui permet de détecter des IgG dirigées contre des antigènes spécifiques et donc de confirmer les résultats obtenus par l’ELISA. L’interprétation des résultats sera optimisée par la discussion entre le clinicien et le biologiste [8]. Pour notre patient, le bilan étiologique habituel de la pancréatite était négatif. En effet, il n’avait pas de prise médicamenteuse, il n’était pas éthylique, le bilan lipidique et la calcémie étaient normaux. Le scanner n’a pas montré de calcul vésiculaire ou de la voie biliaire principale. La toxocarose était découverte à l’occasion de l’hyperéosinophilie récente. En effet le malade n’avait pas d’autres signes orientant vers cette parasitose à part la notion d’élevage de chiens. Le diagnostic a été confirmé par la sérologie qui était positive par la technique ELISA et Western blot. Le diagnostic de toxocarose pancréatique a été suggéré après avoir éliminé les étiologies les plus communes des pancréatites. A notre connaissance, l’association toxocarose et pancréatite n’a pas été décrite dans la littérature ainsi notre observation semble la première qui illustre cette association. Deux hypothèses peuvent être évoquées pour expliquer le mécanisme par lequel la toxocarose entrainerait une pancréatite: la migration du *Toxocara* dans le pancréas et l’infiltration parenchymateuse par les polynucléaires éosinophiles. En effet, l’infiltration pancréatique par les polynucléaires éosinophiles a été rapportée comme cause de pancréatite chronique et plus rarement de pancréatite aigue principalement au cours du syndrome hyperéosinophilique et la gastroentérite à éosinophile [9].

## Conclusion

Bien qu’elle ne soit pas décrite dans la littérature, la recherche de toxocarose devrait faire partie du bilan étiologique de pancréatite aigue lorsqu’elle s’associe à une hyperéosinophilie. Le traitement repose surtout sur l’albendazole.

## Conflits d’intérêts

Les auteurs ne déclarent aucun conflit d'intérêts.
